# Change Regularity of Taste and the Performance of Endogenous Proteases in Shrimp (*Penaens vannamei*) Head during Autolysis

**DOI:** 10.3390/foods10051020

**Published:** 2021-05-08

**Authors:** Shujian Wu, Mouming Zhao, Shijue Gao, Yue Xu, Xiaoying Zhao, Mingyuan Liu, Xiaoling Liu

**Affiliations:** 1 College of Light Industry and Food Engineering, Guangxi University, Nanning 530004, China; sjwu@stu2018.jnu.edu.cn (S.W.); zmmgxu@gxu.edu.cn (M.Z.); gshijue@stu2018.jnu.edu.cn (S.G.); 7200112034@stu.jiangnan.edu.cn (Y.X.); zxy16896@163.com (X.Z.); jacoblmy@outlook.com (M.L.); 2 Department of Food Science and Technology, College of Science & Engineering, Jinan University, Guangzhou 510632, China; 3 School of Food Science and Engineering, South China University of Technology, Guangzhou 510640, China

**Keywords:** shrimp head, autolysis, taste, umami, endogenous proteases

## Abstract

This study evaluated the food safety and proximate composition of shrimp head (SH). Potentially toxic elements in SH were below European Union legislation limits. SH had a high content of tasting amino acids (sweet and umami amino acids was 57%) and a high content of functional amino acids (essential amino acids was 37%). Moreover, the changes of flavor and key umami molecules in SH were studied by sensory evaluation, electronic tongue, electronic nose, automated amino acid analyzer, and high performance liquid chromatography (HPLC). The results showed that the significant difference of flavor in SH happened during autolysis. SH with autolysis had the best umami taste at 6 h, which may result from the synergistic work of free amino acids and nucleotide related compounds. Additionally, the performance of endogenous proteases in SH was investigated to efficiently analyze autolysis. The optimum pH and temperature of endogenous proteases in SH were 7.5 and 50 °C, respectively. The autolysis of SH depends on two endogenous proteases (~50 kDa and ~75 kDa). These results suggest that the formation of flavor in SH during autolysis can be controlled, which could provide guidance for SH recycle. SH could consider as one of the food materials for producing condiments.

## 1. Introduction

Shrimp and shrimp products are widely popular all over the world and their demand is increasing yearly owing to the nutritional characteristics and meat taste. In China, it is estimated that shrimp industries produced over 1,000,000 t of shrimp in 2011 [1]. Generally, shrimp head (SH) is removed in shrimp processing industries, and it accounts for about 35–45% of total shrimp weight [2,3]. It results in more than 300,000 t of SH annually [4]. With the ease of spoilage, SH wastes have been a severe environmental disaster, including waste collection, disposal, and pollution problems. Therefore, there is a need to make use of such wastes in the most efficient manner.

Economical and efficient use of SH would minimize the pollution problem while maximizing the profitability of the processor. SH is a rich source of protein and nutritive components (minerals, carotenoid, etc.) [5,6]. Moreover, some studies reported that the amino acid (AAs) compositions of SH (*Penaeus vannamei*) contribute to a good taste and nutritional value [2,3]. Thus, hydrolysates derived from SH could be used as raw materials in food supplements or flavor enhancers.

There are many endogenous proteases, such as trypsin and chymotrypsin, in the viscera of SH, which lead to degrading tissue proteins by autolysis [2,7,8]. Autolysis is an efficient method to obtain hydrolysates from SH without expensive exogenous enzymes. Meanwhile, the relationship between the formation of flavor and autolysis is a key factor for SH recycle in food products. However, there is little information about the autolysis of SH, especially the influence on flavor. Electronic tongue (E-tongue) and electronic nose (E-nose) are an array of sensors to simulate the tongue and nose of humans, which are efficient analytical tools in food flavor [9]. Therefore, in order to make full use of SH and autolysis, the change regularity of flavor in SH during autolysis was studied by E-tongue and E-nose in this study. Additionally, the free amino acids (FAAs) and nucleotide-related compounds in SH were analyzed during autolysis by an automated amino acid analyzer and HPLC. Moreover, total volatile basic nitrogen (TVB-N) is one of the biomarkers of protein and amine degradation [10], which is measured in seafood to reveal microbiological spoilage [11]. We determined the level of TVB-N in SH to ensure its food safety. On the other hand, the reaction times and efficiency of autolysis are affected by the kinds of endogenous proteases and autolysis conditions that included temperature, pH, etc. The performance of endogenous proteases was further investigated in the present study. The aim of this study was to research an effective and low-cost autolysis method for SH recycle, which can provide guidance in the shrimp industry through reasonably control autolysis.

## 2. Materials and Methods

### 2.1. Materials and Chemicals

SH was obtained by Guangxi Zhengwu Marine Industry Co., Ltd. (Beihai, China). Fresh SH was stored on ice with clean sanitized containers after obtaining them from the whole shrimp, which was transported to the laboratory and processed immediately.

The standards of the nucleotide flavor compound were purchased from Shanghai Ocean Biotechnology Co., Ltd. (Shanghai, China), which were used to make a standard curve for the determination of nucleotide flavor compounds in SH. Methanol was of HPLC grade. Other chemicals were only used for analytical grade reagents.

### 2.2. Determination of Pb, Cd, Hg, As, and Proximate Composition

Toxic elements (Pb, As, Hg, and Cd) in SH were determined by ICP–AES. The method was described in detail by Albuquerque et al. [12]. The SH was dried at 105 °C for 24 h to determine moisture content, the protein was determined by the Kjeldahl method, and the SH was incinerated at 550 °C for the determination of ash.

### 2.3. Autolysis of SH

The autolysis of SH was conducted by the method of Cao et al. [2], with some modifications. The fresh SH was ground by a stirrer (JYL-C022E, Joyoung Co., Ltd., Shandong, China). After that, the SH were placed into a sterilized beaker and put into a water bath at 25 °C to autolysis (simulated ambient temperature). The reaction was terminated after the reaction up to 10 h.

### 2.4. Determination of TVB-N

The determination of TVB-N was according to in accordance with the Chinese Standard GB 5009.228-2016 [13]. The analysis includes a perchloric acid extraction, followed by alkalization and steam distillation by Kjeldahl Instrument (SKD.600, Peiou Analysis Instrument Co., Ltd., Shanghai, China). The boric acid solution was used to absorb the total volatile bases. After that, the TVB-N value was titrated with a hydrochloric solution.

### 2.5. Determination of AAs

The composition of AAs was measured through a fully automated amino acid analyzer (L-8900, Hitachi, Japan) by the method described by Cao et al. [2]. Briefly, samples (2 g) were added into 6 mol/L HCl (10 mL). The mixture was sealed and degraded at 110 °C for 22 h. After that, the hydrolysate was filtered by a filter paper, and then the solution was mixed with ultrapure water to a final volume of 50 mL. A total of 1 mL of the sample solution was added to a 10 mL volumetric flask in a water bath at 60 °C to remove HCl, and then the solution was mixed with ultrapure water to 10 mL. The sample was filtered via a 0.22 μm filter before determination.

FAAs were analyzed by the method of Dabadé et al. [14]. Sample (5 g) and 15 mL water were added to the beaker and stored at 4 °C for 15 min. Then, 10% trichloroacetic acid (TCA, 15 mL) was added to scale to precipitate the peptide or protein, followed by incubation at 4 °C for 15 min, and then centrifuged at 10,000 r/min for 10 min (5418R, Eppendorf, Germany). The supernatant was diluted to 250 mL in a volumetric flask with 10% TCA. The sample was filtered via a 0.22 nm filter and stored at 4 °C before assay.

### 2.6. Determination of Nucleotide-Related Compounds

The determination of nucleotide-related compounds was modestly modified according to the methods reported by Zhang et al. [15]. The homogenized SH and ultrapure water were added into a beaker 1:3 (g:mL) and then stored at 4 °C for 30 min with a magnetic stirrer. The mixture was centrifuged at 6000 r/min (4 °C, 10 min) to obtain a supernatant. After that, the supernatant (5 mL) and 8% perchloric acid (15 mL) were added into a beaker and stored at refrigerator (4 °C,10 min), then centrifuged at 6000 r/min (4 °C, 10 min). The supernatant was collected. The precipitate was repeated again and pooled the supernatants. Afterward, the pH of supernatants was adjusted to 6.5 by KOH with different concentrations. The neutralized supernatant was diluted to 100 mL with neutralized perchloric acid (pH 6.5). The sample was filtered via a 0.22 nm filter. The resulting solution was analyzed by HPLC (Waters e2695, Milford, MA, USA) and UV/Visible Detector (Waters 2489, Milford, MA, USA).

The column was an Agilent C18 column (4.6 mm × 250 mm, 5 μm). The Column temperature was 25 °C, and the injection volume was 10 μL. Additionally, mobile phase: A, KH_2_PO_4_ / K_2_HPO_4_ buffer (pH 6.5); B, methanol. All solvents were filtered and degassed before use. Detection at 254 nm. Speed: 0.7 mL/min. Gradient of elution: 0–6 min, 98% A and 2% B; 6–10 min, B increased to 5%, A reduced to 95%; 10–14 min, B increased to 15%, A reduced to 85%; 14–18 min, B reduced by 10%, A increase to 90%; 18–25 min, B reduction of 5%, B increased to 95%. The column was equilibrated for 5 min before the next sample.

### 2.7. Sensory Evaluation

The sensory evaluation was the method of Yu et al. [16] and the Chinese Standard (GB/T 12312 and GB/T 12315) [17,18]. All panelists (eight members, half males and half females) were screened by Chinese Standard GB/T 12312 (Sensory analysis—Methodology—Method of investigating the sensitivity of taste, MOD) [17]. These people could recognize the umami solution and had received training in descriptive sensory analysis. The homogenized SH and pure water were mixed at the final substrate concentration to 1:3 (g:mL) and stored at 4 °C for 20 min with a magnetic stirrer. The mixture was centrifuged at 8000 r/min for 10 min and obtain the supernatant. The soluble solids content of the supernatant was adjusted 1% (Sugar Refrectometer, WZS, Jingke, China) with pure water for sensory analysis. Sensory evaluation was a ranking test according to the Chinese Standard GB/T 12315 (Sensory analysis—Methodology—Ranking) [18]. Every sample was randomly numbered before the experiment to hide its information. Every panelist ranked the samples according to the intensity of umami taste (from 1 to 6, no same rank). The experiment was carried out in an air-conditioned room (25 ± 2 °C), and panelists were separated in each booth. The results were analyzed by the Friedman test [18].

(1) $$F{\;}_{\mathrm{test}}=\frac{12}{j.p\;(p+1)}({R^{2}}_{1}+\ldots +{R^{2}}_{x}+{R^{2}}_{y})-3j(p+1)$$

(2) $$\mathrm{Least}\;\mathrm{Significant}\;\mathrm{Difference}\;(\mathrm{LSD})=z\sqrt{\frac{j.p(p+1)}{6}}$$

where F _test_ > F means the significant difference among all samples; R*_x_* – R*_y_* > LSD means the significant difference between the sample of *x* and the sample of *y*. *j* = the number of panelists; *p* = the number of samples; R_1_ = the rank sum of the sample, which was ranked first in all samples, etc. to R*_x_*_,_ R*_y_*; the value of *z* and F were 1.96 and 10.68, respectively, when *j* = 6 and *p* = 8, which were obtained from the table of Friedman test according to the Chinese Standard GB/T 12315.

### 2.8. E-tongue Analysis

E-tongue analysis was performed by TS-5000Z (Insent, Kanagawa, Japan). Sample preparation was consistent with sensory evaluation. The experiment was determined by the method of Zhu et al. [19]. A 50 mL sample was put into a cup. The measure progress was “maintenance measurement.” Four replicates were completed for each group and then retained three stable sets of data. The detailed information of five chemical sensors in E-tongue is in Table S1.

### 2.9. E-nose analysis

The experiment was according to the method from Zhu et al. [19] (PEN3, Germany). Briefly, a 15.00 g homogenized sample was placed into a 150 mL sterilized beaker and sealed the top. Then, the sample was equilibrated (10 min, 25 °C) to minimize sensor drift due to environmental changes [19]. The flush time, presampling time, and measurement time were 60 s, 5 s, and 70 s, respectively. Every sample was determined three times. Four replicates were completed for each group and then retained three stable sets of data. the statistically significant difference (*p* < 0.05) in the mean of each sensor was obtained by the least significant difference (LSD) test [19,20]. The detailed information of the 10 chemical sensors in E-nose is in Table S2.

### 2.10. Determination of pH Value

The pH of homogenized SH was determined based on the method from Shi et al. [21].

### 2.11. Determination of Endogenous Enzyme Activity

The determination of endogenous enzyme ratio activity was determined by the method from Hang et al. [22]. Briefly, 4 mL of Tris-HCl buffer (pH 7, 0.05mol/L) and 1.6mL of 1% casein solution were placed into two test tubes, numbered I and II, respectively. Then, 2.4 mL of 10% TCA was added in I (as control, to inhibit the action of enzymes), and the two test tubes were put into a water bath (35 °C) for 5 min. After that, a 0.8 mL sample was added into I and II and incubated at 35 °C for 15 min. Next, 10% TCA (2.4 mL) was added in II. Finally, the resulting solution was centrifuged at 10,000 r/min for 15 min to obtain the supernatant, of which the absorbance was measured by UV-6100 spectrophotometer (Shanghai Meipuda Instrument Co., Ltd. Shanghai, China) at 275 nm. One unit (U/mL) of enzyme activity was defined as the amount of enzyme capable of hydrolyzing casein to produce a 0.001-unit change in absorbance per minute [23].

(3) $$a\;\left(U/\mathrm{mL}\right)=\frac{\Delta \mathrm{OD}}{15\min\times 0.001\times 0.8\;\mathrm{mL}}$$

Different pH for extraction of SH crude extract (CE) was determined in our preliminary research (Figure S1). the optimal pH to obtain CE was 7.5. The homogenized SH and Tris-HCl buffer was added into a beaker (1:4 (g:mL)) and stored at 4 °C with a magnetic stirrer (5 min). The solution was centrifuged at 8000 r/min for 10 min to obtain a supernatant for future experiments.

### 2.12. Separation of Enzyme

The CE was precipitated with saturated ammonium sulfate solution (30%) at 4 °C for 1 h. The supernatant was obtained from the mixture by centrifuged at 10,000 r/min (4 °C, 15 min). Afterward, the supernatant was precipitated with saturated ammonium sulfate solution (60%) at 4 °C for 1 h. The precipitate was obtained by centrifuged at 10,000 r/min (4 °C, 15 min). The precipitate was redissolved with three times volume of Tris-HCl buffer, which was crude endogenous proteases (CEP). The CEP was further separated by DEAE-Sepharose FF column chromatography. The column temperature was 25 °C, and injection volume was 5 mL. The detection wavelength was 280 nm. Collection: 10 min/tube. Speed: 1 mL/min. Gradient of elution by NaCl with different concentration (first, 80 mL, 0.25 mol/L; then, 80 mL, 0.50 mol/L; final, 80 mL, 1 mol/L).

(4) $$\mathrm{Enzyme}\;\mathrm{ratio}\;\mathrm{activity}\;\left(U/\mathrm{mg}\;\mathrm{protein}\right)=\frac{\mathrm{Enzyme}\;\mathrm{activity}\;\mathrm{of}\;\mathrm{sample}}{\mathrm{Protein}\;\mathrm{content}\;\mathrm{of}\;\mathrm{sample}}$$

(5) $$\mathrm{Recovery}\;\mathrm{rate}\;\mathrm{of}\;\mathrm{enzyme}\;\mathrm{activity}\;\left(\%\right)=\frac{\mathrm{Total}\;\mathrm{enzyme}\;\mathrm{activity}\;\mathrm{of}\;\mathrm{sample}}{\mathrm{Total}\;\mathrm{enzyme}\;\mathrm{activity}\;\mathrm{of}\;\mathrm{CE}}\times 100$$

(6) $$\mathrm{Purification}\;\mathrm{factor}=\frac{\mathrm{Enzyme}\;\mathrm{ratio}\;\mathrm{activity}\;\mathrm{of}\;\mathrm{sample}}{\mathrm{Enzyme}\;\mathrm{ratio}\;\mathrm{activity}\;\mathrm{of}\;\mathrm{CE}}$$

### 2.13. SDS–PAGE

The SDS–PAGE was based on the method described by Beloborodov et al. [24]. A total of 20 μL sample and 80 μL 5 × SDS–PAGE loading buffer were mixed in a tube and then put into boiling water bath for 10 min. The separation gel was 12% polyacrylamide gel.

### 2.14. Statistical Analysis

The experimental data were analyzed by SPSS 19.0 (SPSS Corporation, Chicago, IL, USA) with a one-way analysis of variance (ANOVA) and the Duncan procedure between means. Ranking data of the sensory evaluation were analyzed using the Friedman test.

## 3. Results

### 3.1. Food Safety and Proximate Composition of SH

**Figure 1:**
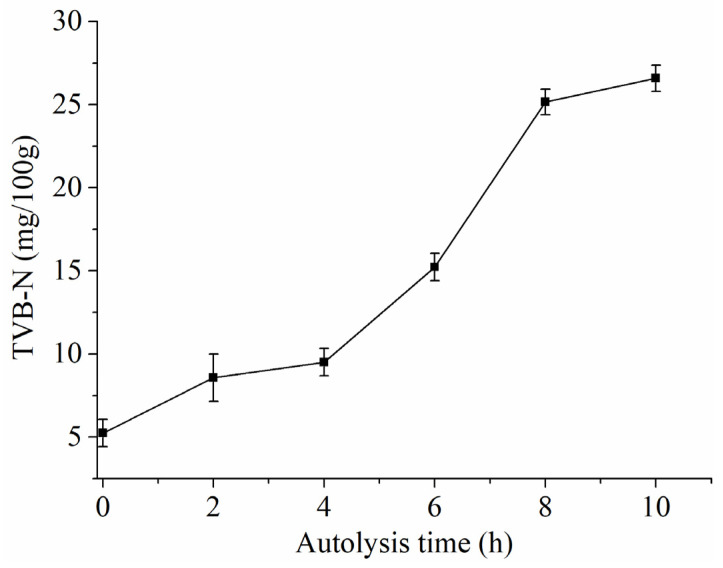
Changes of TVB-N in shrimp head during autolysis.

Table 1 shows that that the concentrations of Pb, Cd, and Hg were below the maximum level of seafood, which was set by the European Commission legislation (ECR) No 1881/2006 and amendments [25]. The concentration of As was not detected. These data indicated that the SH complied with the standard of food safety. Table 2 shows that the moisture content, ash, and protein were 77.47% ± 0.05%, 4.47% ± 0.01%, and 10.32% ± 0.09%, respectively, in SH, which suggested that SH is one of the rich sources for proteins. Additionally, the inedible range of TVB-N for raw shrimps is >30 mg/100 g in the standard of China [26]. As Figure 1 shows, the accepted range of TVB-N for SH is autolysis within 10 h (26.37 mg/100 g). Thus, we studied the change regularity of flavor in SH within 10 h.

The composition of AAs is a key role in SH when it is used as a raw material for condiments. As shown in Table 3, the SH almost meets the FAO/WHO (1973) requirements that essential amino acids (EAAs) and the value of the ratio in EAAs to nonessential amino acids (NEAAs) are 40% and 0.6 in foods, respectively [27,28]. The EAAs and EAAs/NEAAs were 37% and 0.57 in SH, respectively. Furthermore, there is an abundance of total sweet and umami amino acids (SUAAs, 57%) in SH. Therefore, SH is one of the good materials to produce condiments.

### 3.2. Change Regularity of Taste in SH during Autolysis

**Figure 2:**
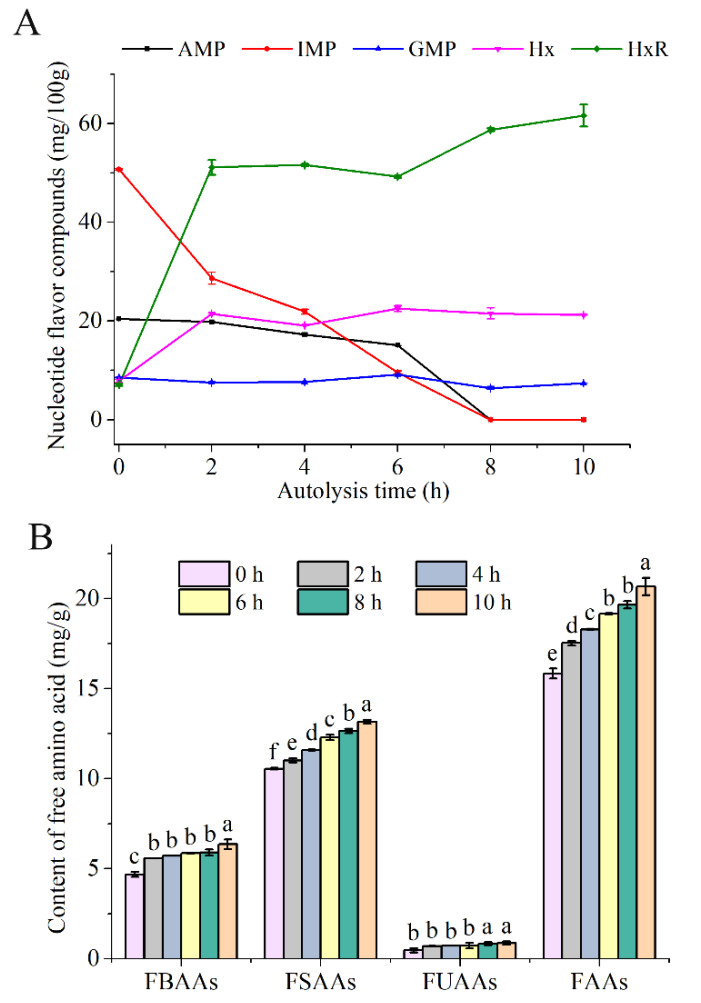
The change of different (**A**) FAAs and (**B**) nucleotide flavor compounds in SH during autolysis. The data marked by different letters means significantly different (*p* < 0.05) in each group. FBAAs, total free bitter amino acids; FSAAs, total sweet amino acids; FUAAs, total free umami acid; FAAs, total free amino acids. Umami amino acids including aspartic acid and glutamic acid. Sweet amino acids including threonine, serine, proline, glycine, methionine, alanine. Bitter amino acids including isoleucine, valine, leucine, tyrosine, methionine, phenylalanine, lysine, histidine, arginine.

One of the important parts of the taste of shrimp and shrimp products is umami taste [19,29]; hence, we mainly focused on the umami change of taste in SH during autolysis. The SH was evaluated and recognized by panelists during autolysis. The results showed that the flavor of SH had the best umami taste at 6 h and 8 h during autolysis (Table 4), but some panelists taste a fishy smell in SH with autolysis at 8 h during sensory evaluation. Generally, FAAs and nucleotide-flavor compounds are key umami molecules in foods, which had the ability to improve the flavor of the sample [30,31,32]. There are umami, sourness, sweetness, saltiness, and bitterness taste in AAs, which contribute to increasing the flavor in food [33]. The major flavor enhancer nucleotides, such as inosine monophosphate (IMP), adenosine monophosphate (AMP), and guanosine monophosphate (GMP), can present umami taste at low levels [34]. Inosine (HxR) and hypoxanthine (Hx) can present bitterness [15]. AMP, IMP, GMP, HxR, and Hx are from the degradation of ATP during autolysis in aquatic products, which plays an important role in the changes of flavor [34,35]. Therefore, we determined the change of these compounds during autolysis. The bitter amino acids (FBAAs), Hx, and HxR were significantly increased in the early stages of autolysis (Figure 2), which may result in off-flavor. The interaction between Hx and certain AAs can also result in a bitter taste [36]. However, the flavor was influenced by the synergistic work of different flavor components. The decreased trend of AMP and IMP was observed during autolysis, even completely degraded at 8 h, while the sweet amino acids (FSAAs) were increased (Figure 2). The umami taste can be improved by the synergistic effect of IMP and FSAAs (e.g., alanine, serine, and glycine) [30,37]. Compared with a single compound, The synergistic effect of some AAs and GMP, adenosine diphosphate (ADP), and AMP express greater umami taste [32]. At 6 h, the FBAAs and HxR were not further increased, but the FSAAs were increased. There are also AMP, GMP, and IMP. After 8 h, a significant increase of FBAAs and HxR and the disappearance of AMP and IMP were observed, which may be one of the reasons for the decrease of umami taste in SH (Figure 2). Thus, SH had good umami taste in autolysis at 6 h, which may explain the umami taste results of sensory evaluation.

### 3.3. E-Tongue and E-Nose Response Signal of SH during Autolysis

**Figure 3:**
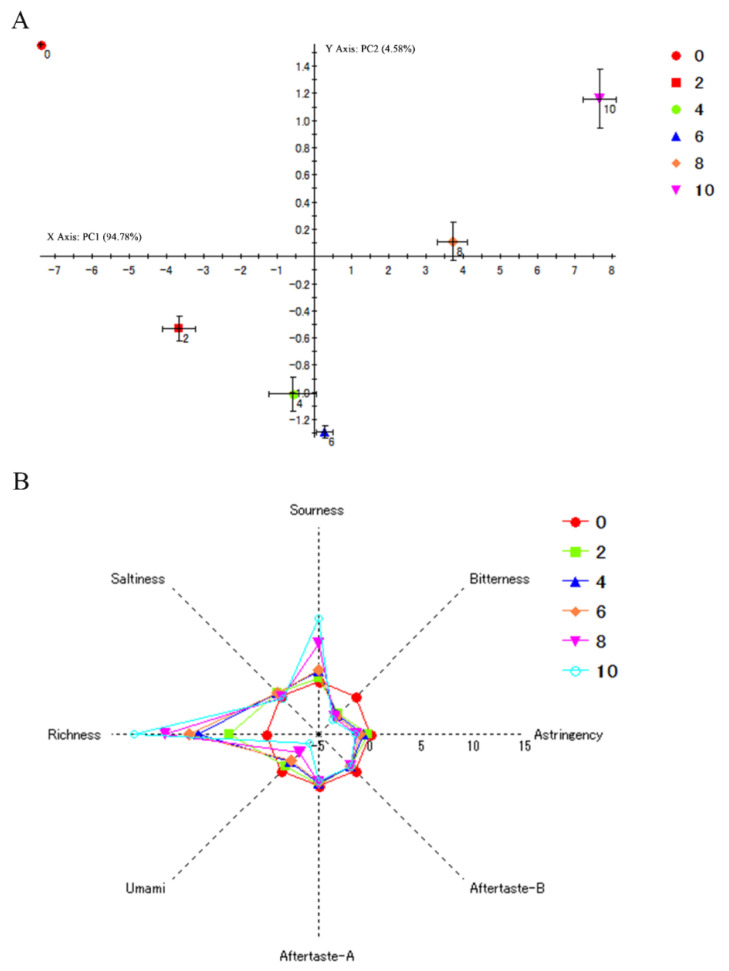
The analysis of shrimp head by E-tongue. (**A**) The principal component analysis of E-tongue; (**B**) The Radar chart analysis of E-tongue.

**Figure 4:**
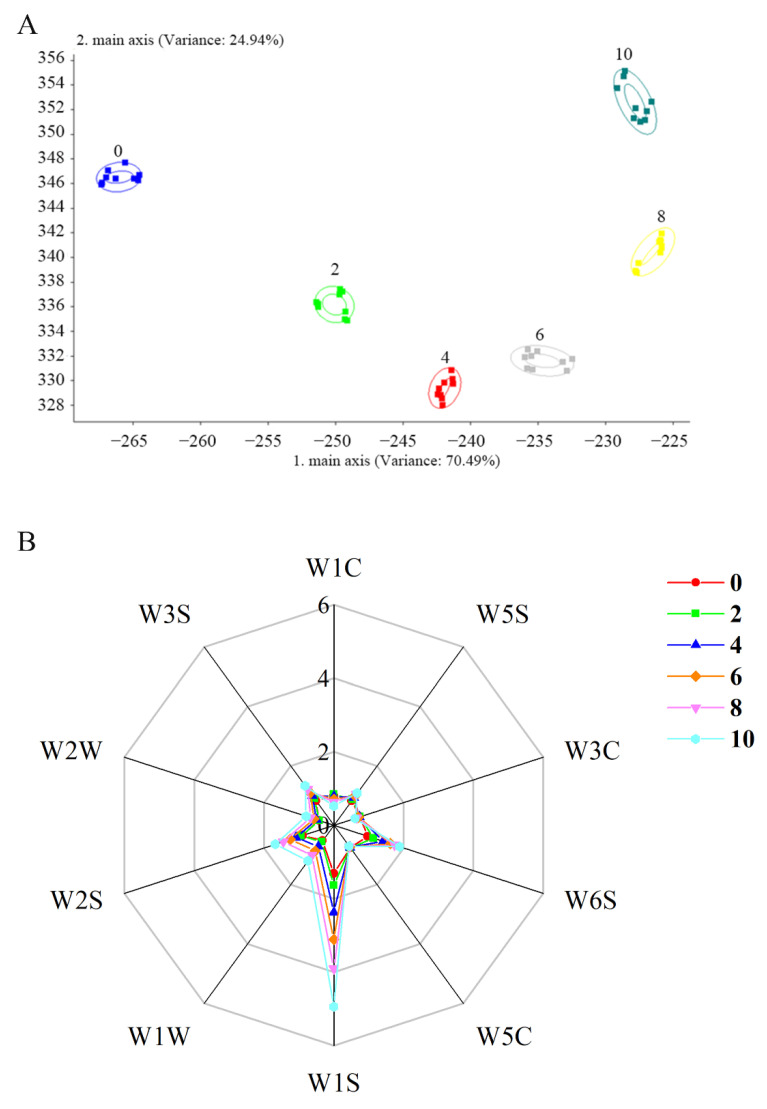
The analysis of shrimp head by E-nose. (**A**) the linear discriminant analysis of E-nose; (**B**) The Radar chart analysis of E-nose.

**Figure 5:**
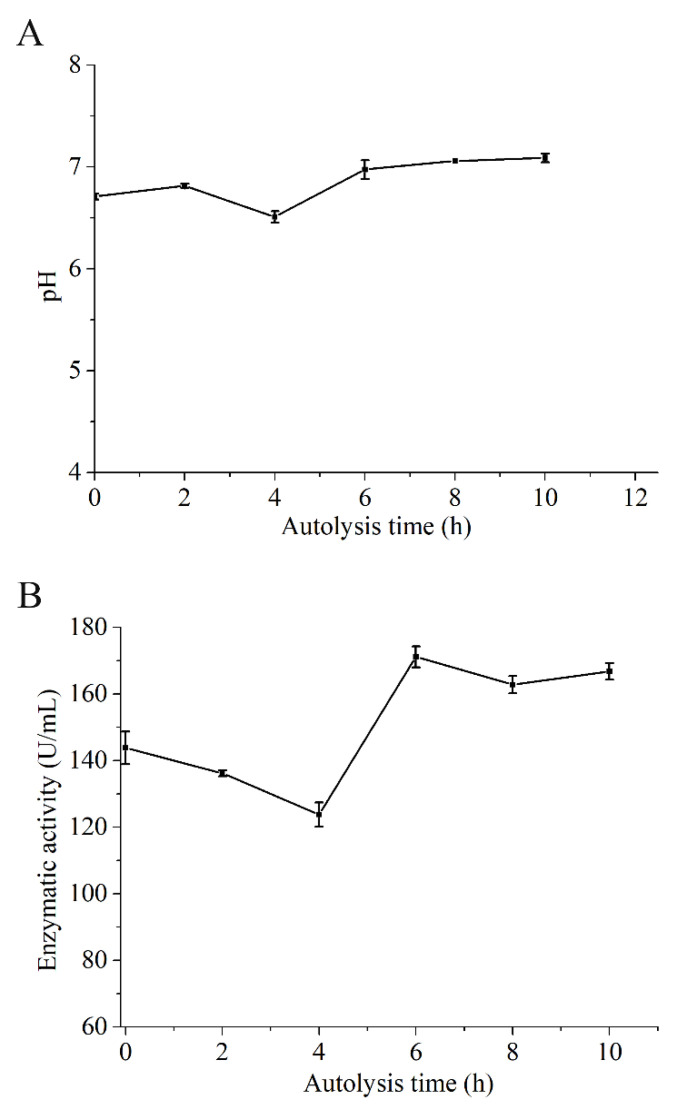
The changes of (**A**) pH and (**B**) enzymatic activity in shrimp during autolysis.

In order to evaluate the changes of taste characterizations during autolysis, SH with autolysis was analyzed by the E-tongue and E-nose. The principal component analysis (PCA) of E- tongue and the linear discriminant analysis (LDA) of E-nose are shown in Figure 3A and Figure 4A, respectively. The total contribution rate of PCA and LDA was 99.36% (PC1, 94.78%; PC2, 4.58%) and 95.43% (LD1, 70.49%; LD2, 24.94%), respectively, which indicated that PCA and LDA can be used to reflect the changes of flavor with a large amount of information in SH during autolysis [38,39]. The data suggested that the differentiation of the groups was good. Thus, the difference of flavor in SH during autolysis can be distinguished and sensed by artificial senses (E-tongue and E-nose). On the other hand, the sample of 4 h and 6 h were close to each other and located further from point 8 h (Figure 3A), which suggested that taste compounds may be similar in samples of 4 h and 6 h, while they were different in the sample of 8 h. According to the results of enzymatic activity (Figure 5B), the enzymatic activity reached lowest at 4 h and then gradually increased. A higher level of enzymatic activity was maintained after 6 h. Thus, the more taste compound may be produced due to the enhancement of the autolysis rate after 6 h, which lead to the significant difference in taste in SH. These results implied there was a significant difference in flavor in SH during autolysis. The result of E-tongue and E-nose have consistency with the sensory evaluation results.

The response values of E-tongue are shown in Figure 3B. Sensor response output of 0 h was calculated as ‘”0” in this study. If the change in concentration of taste substance exceeds 1.0 scale, the human tongue can recognize the difference of sample [19,40]. With the increase in time of autolysis, the richness and sourness of SH increased, and the bitterness and umami taste decreased. No marked differences in saltness, astringency, aftertaste A, and aftertaste B were observed. Interestingly, there was inconsistency between the decrease of umami and bitterness and the increase of FBAAs and FUAAs in SH during autolysis (Figure 2). These data implied that the taste is a comprehensive sense by the synergistic work of different flavor components. The mechanism among amino acids, E-tongue, and taste should be studied in future work, which may usefully guide the development of food products. Additionally, according to the results of pH (Figure 5A), the production of acids may cause the enhance of sourness. Humans accept sour taste when mild but reject when strong [41]. The sourness increased mildly within 6 h, while increased sharply after 6 h, which may help to explain the result of sensory evaluation. Richness defines flavor intensity. The enhance of richness in SH suggested that the changes of taste are obvious and can be distinguished during autolysis. The reduction in bitterness may be due to the enhance in free sweet amino acids, which can mask bitter tastes. In summary, the changes of umami in SH may be caused by the combined work of sourness, richness, bitterness.

As the radar chart in Figure 4B shows, the odor characteristics were significantly different in SH during autolysis. The response values of sensors W1S (sensitive to methane), W2S (sensitive to ethanol), W1W (sensitive to sulfides), and W6S (mainly selective for hydrogen) differed significantly among all samples, whereas the response values of W1C, W3S, W5S, W2W, W3C, and W5C were similar. The response values of W1S, W2S, and W6S were enhanced during the extension of autolysis, which indicated methyl, aldehydes, ketones, and hydrides were increased in SH. The response value of W1W was decreased with the extension of autolysis, which indicated sulfur compounds were decreased in SH. Thus, autolysis time had a significant influence on methyl, aldehydes, ketones, hydrides, and sulfur, which may play an important role in the umami of SH.

### 3.4. Change Regularity of pH and Enzymatic Activity in SH during Autolysis

pH is closely related to the umami flavors. Feng et al. [42] found the umami of six umami additives were significant affected by pH from 5 to 8. The best working pH for most umami substances was 6–7 [42,43]. Meanwhile, endogenous protease plays a key role in the degradation of protein and nucleotide-flavor compounds [44]. The changes in pH and enzymatic activity of SH during autolysis were studied (Figure 5). At the early stage of autolysis, the anaerobic glycolysis and the digestion of ATP were increased. pH decreased from 6.71 to 6.51 [45]. Afterward, microbial metabolites (such as TVB-N and trimethylamine-nitrogen) were accumulated rapidly with the activation of endogenous protease (Figure 1 and Figure 5B), which are the main reason for pH increment to 6.97 [46,47] and becoming stabilized thereafter. Feng et al. [42] found that the umami of umami flavoring was increased with the increase of pH within 6.5–7.0, which could help to explain the change of taste in SH (Table 4). In addition, the trend of change in enzyme activity was similar to pH (Figure 5). The native protein of SH could be degraded rapidly by endogenous protease in digestive organs during autolysis [44]. Therefore, the total free amino acids were increased in SH (Figure 2). However, there may be more off-flavor compounds with the further activation of endogenous protease, including FBAAs, Hx, and HxR (Figure 2), which lead to the unpleased flavor in SH (Table 4). The results indicated that the autolysis time needs to be controlled at a suitable time to obtain a good flavor.

### 3.5. Characteristics of Endogenous Protease of SH

**Figure 6:**
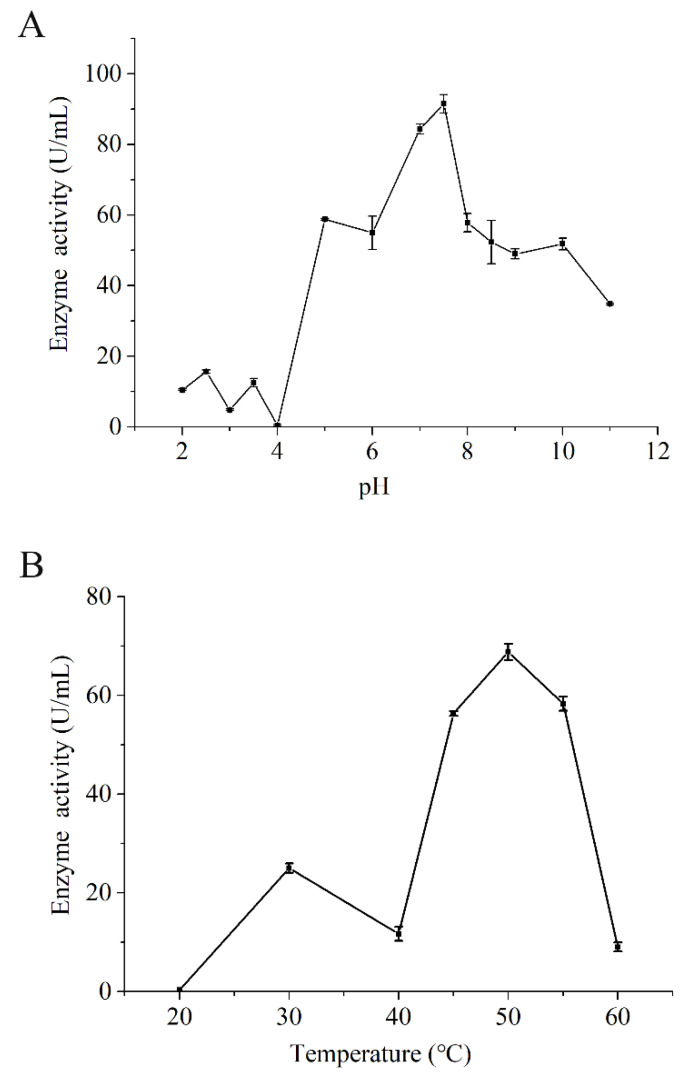
The effects of (**A**) pH and (**B**) temperature for the endogenous protease.

**Figure 7:**
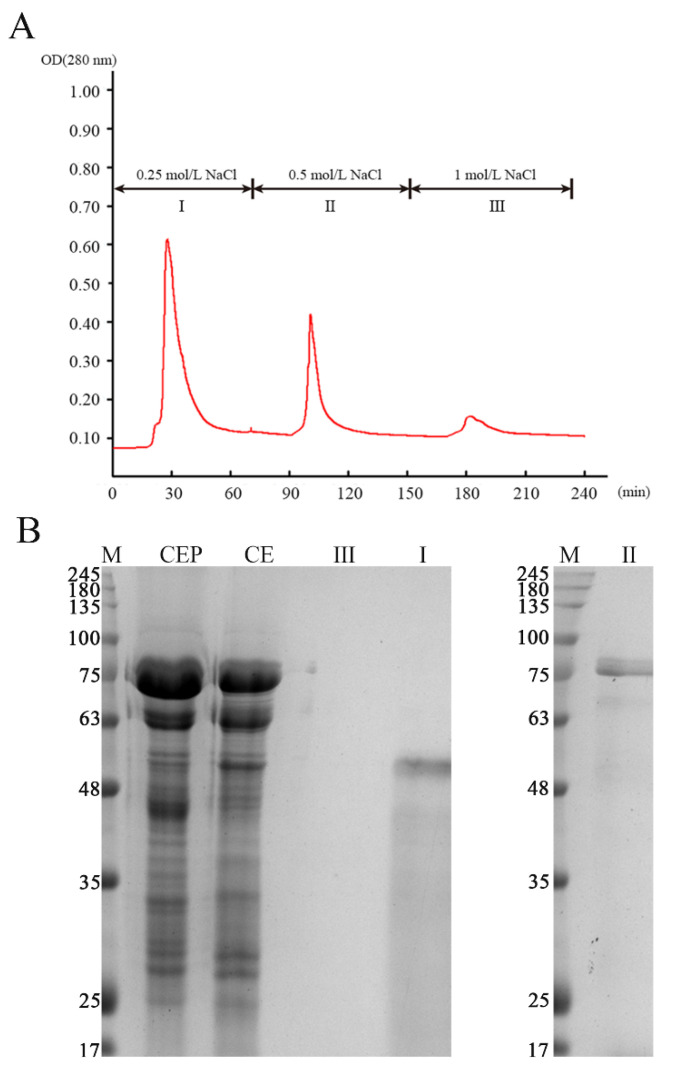
The purification of endogenous protease from shrimp head: (**A**) endogenous proteases were separated and purified by DEAE–Sepharose fast flow and (**B**) the SDS–PAGE analysis of endogenous protease from shrimp head. CE, SH crude extract; CEP, crude endogenous protease.

The autolysis of SH depends on different physicochemical conditions, including the temperature of incubation and pH. The influence of different temperatures and pH on enzyme activity of CEP was evaluated (Figure 6). The maximum activity under the assayed conditions took place at 50 °C and pH 7.5, respectively, which may be the optimal conditions to utilize autolysis. this result was similar to the results of Cao et al. [3] (50 °C and pH 7.85). The CEP was further separated and purified by DEAE–Sepharose fast flow column chromatography, providing three fractions (I, II, and III; Figure 7A). The outline and the results of the separation and purification of the endogenous protease are summarized in Table 5. The results indicate that I and II were the main endogenous protease of SH, especially II. SDS–PAGE analysis showed endogenous protease contained two major enzymes (~50 kDa and ~75 kDa, Figure 7B) which were most likely responsible for the changes of flavor during autolysis.

## 4. Conclusions

In summary, SH could be one of the sources for condiments with a good composition of AAs, and it complies with the requirement of food safety. Moreover, there was a significant difference in flavor in SH during autolysis, which may be caused by the synergistic work of FAAs and nucleotide-related compounds. SH with autolysis had the best umami taste at 6 h. Meanwhile, the optimal conditions to utilize autolysis may be at 50 °C and pH 7.5. Two endogenous proteases (~50 kDa and ~75 kDa) play a key role in autolysis. Thus, it is a good economic use of SH to prepare umami hydrolysates from SH by the reasonable control of autolysis, which can be as a raw material in food supplements. The SH recycle with autolysis is an efficient way to solve the problems of SH waste, which complete the low-cost and high-value utilization in by-products of the shrimp industry. The autolysis also can be used for the development of food products and the recycling of chitin, lipid, and astaxanthin in SH. Furthermore, the results of sensory evaluation, E-tongue, and E-nose are consistent, which implied that a combination of these analytical techniques can be applied to study the formation of flavor to obtain more comprehensive and accurate results.

## Figures and Tables

**Table 1:** The concentrations of Pb, As, Cd, and Hg in SH (dry basis, mg/kg).

Toxic Elements	Limit of ECR	Concentrations in SH
Pb	≤0.5	0.03
As	Not established	Not detected
Cd	≤0.5	0.26
Hg	≤0.5	0.01

**Table 2:** The concentrations of proximate composition in SH (wet basis, %).

Moisture Content	Crude Ash	Protein
77.47 ± 0.05	4.47 ± 0.01	10.32 ± 0.09

**Table 3:** Composition of amino acids in shrimp head (wet basis, mg/g).

Amino Acid	Content
Glycine	7.62 ± 0.08
Alanine	5.86 ± 0.02
Histidine	2.32 ± 0.19
Tyrosine	4.68 ± 0.55
Serine	3.95 ± 0.05
Cysteine	0.40 ± 0.05
Aspartic acid	8.23 ± 0.08
Glutamic acid	15.46 ± 0.17
Arginine	8.98 ± 0.11
Proline	6.75 ± 0.05
Leucine	6.98 ± 0.08
Isoleucine	3.77 ± 0.03
Valine	4.35 ± 0.04
Threonine	3.77 ± 0.04
Methionine	5.18 ± 0. 34
Phenylalanine	5.28 ± 0.51
Lysine	7.20 ± 0.26
Total amino acids (TAAs)	100.83 ± 1.68
Essential amino acids (EAAs)	36.55 ± 0.54
Nonessential amino acids (NEAAs)	64.27 ± 1.15
Sweet and umami amino acids (SUAAs)	56.84 ± 0.43
SUAAs/TAAs	57%
EAAs/TAAs	37%
EAAs: NEAAs	0.57

Umami amino acids including aspartic acid, glutamic acid; sweet amino acids including threonine, serine, methionine, glycine, proline, alanine.

**Table 4:** Ranking test results of shrimp heads in autolysis on umami taste.

Autolysis Time (h)	0	2	4	6	8	10
Sum of ranks	39 ^c^	40 ^c^	36 ^b,c^	15 ^a^	15 ^a^	23 ^a,b^

The data marked by different letters are significantly different (*p* < 0.05).

**Table 5:** A summary of the purification of endogenous protease on the extract from shrimp heads.

Purification	Enzymatic Ratio Activity (U/mg Protein)	Recovery of Enzymatic Activity (%)	Purification Fold
Crude extract	2.93	100	1
Crude endogenous protease	4.16	89.13	1.42
I	3.22	15.12	1.10
II	6.54	60.88	2.23
III	Not detected	-	-

## Supplementary Materials

The following are available online at https://www.mdpi.com/article/10.3390/foods10051020/s1, Figure S1: Changes of enzyme ratio activity of the crude extract were extracted from shrimp head in different pH buffer, Table S1: The information of chemical sensors in E-tongue, Table S2: The information of chemical sensors in E-nose.
