# Supplementary material for: Change Regularity of Taste and the Performance of Endogenous Proteases in Shrimp (Penaens vannamei) Head during Autolysis
Source: Foods. 2021 May 8;10(5):1020. doi: 10.3390/foods10051020 (PMC8151679; doi:10.3390/foods10051020)
Supplement: Supplementary file 1 [file foods-10-01020-s001.zip › Table S1.pdf]

Table S1. The information of chemical sensors in E-tongue

Chemical Sensor	Determination of taste
CA0	sourness
AE1	astringency
AAE	umami sensor
CT0	saltiness
C00	bitterness
