# Supplementary material for: Change Regularity of Taste and the Performance of Endogenous Proteases in Shrimp (Penaens vannamei) Head during Autolysis
Source: Foods. 2021 May 8;10(5):1020. doi: 10.3390/foods10051020 (PMC8151679; doi:10.3390/foods10051020)
Supplement: Supplementary file 1 [file foods-10-01020-s001.zip › Table S2.pdf]

Table S2. The information of chemical sensors in E-nose

Chemical Sensor	Determination of volatile substances
W1C	Aromatic ingredients
W5S	High sensitivity and sensitive to nitrogen oxides
W3C	Ammonia, sensitive to aromatic ingredients
W6S	Mainly selective for hydrogen
W5C	Alkane aromatic component
W1S	Sensitive to methane
W1W	Sensitive to sulfides
W2S	Sensitive to ethanol
W2W	Aromatic ingredients, sensitive to organic sulfides
W3S	Sensitive to alkanes
